# Monitoring progression of clinical reasoning skills during health sciences education using the case method – a qualitative observational study

**DOI:** 10.1186/s12909-017-1002-4

**Published:** 2017-09-11

**Authors:** Kristina Orban, Maria Ekelin, Gudrun Edgren, Olof Sandgren, Pia Hovbrandt, Eva K. Persson

**Affiliations:** 1 Department of Health Sciences, Lund University, P.O. Box 157, SE-221 00 Lund, Sweden; 2 Faculty of Medicine Centre for Teaching and Learning, Lund University, P.O. Box 157, SE-221 00 Lund, Sweden; 3 Logopedics, Phoniatrics, & Audiology, Department of Clinical Sciences, Lund University, P.O. Box 117, SE-221 00 Lund, Sweden

**Keywords:** Clinical problem-solving, Professional development, Health sciences education, Qualitative content analysis

## Abstract

**Background:**

Outcome- or competency-based education is well established in medical and health sciences education. Curricula are based on courses where students develop their competences and assessment is also usually course-based. Clinical reasoning is an important competence, and the aim of this study was to monitor and describe students’ progression in professional clinical reasoning skills during health sciences education using observations of group discussions following the case method.

**Methods:**

In this qualitative study students from three different health education programmes were observed while discussing clinical cases in a modified Harvard case method session. A rubric with four dimensions – *problem-solving process, disciplinary knowledge, character of discussion* and *communication* – was used as an observational tool to identify clinical reasoning. A deductive content analysis was performed.

**Results:**

The results revealed the students’ transition over time from reasoning based strictly on theoretical knowledge to reasoning ability characterized by clinical considerations and experiences. Students who were approaching the end of their education immediately identified the most important problem and then focused on this in their discussion. Practice knowledge increased over time, which was seen as progression in the use of professional language, concepts, terms and the use of prior clinical experience. The character of the discussion evolved from theoretical considerations early in the education to clinical reasoning in later years. Communication within the groups was supportive and conducted with a professional tone.

**Conclusions:**

Our observations revealed progression in several aspects of students’ clinical reasoning skills on a group level in their discussions of clinical cases. We suggest that the case method can be a useful tool in assessing quality in health sciences education.

## Background

Outcome-based, or competency-based, education has been an emerging trend in medical and health sciences education for decades, presented as a better alternative to older content- and time-based curricula [1–3]. In Europe, the Bologna agreement of 1999 [4] started a movement towards increased comparability in higher education. Frameworks for qualification as learning outcomes formed an important part of the process [5] as well as standardisation of credits. Assessment of competencies constitutes an important part of those curricula for summative assessment of individuals as well as for feedback to teachers about the quality of the curriculum. For both these purposes, it is important to assess students’ development towards their future profession during the course of study [6]. Both students and teachers require frequent information on how the students are proceeding towards specified outcomes/competencies. Progress tests are commonly used [7–10] and are usually multiple-choice tests given simultaneously for all cohorts in the education programme. In engineering education, students’ professional skills have been monitored using observation of teams while solving problems [11]. To our knowledge, progression in clinical reasoning skills, in health sciences have not previously been evaluated by using the case method. Inspired by the study by Wahlgren and Ahlberg [11], we undertook to use clinical cases to monitor progression and to evaluate, using standardized criteria, how students in health professions develop professional clinical reasoning skills. If progression can be identified this method may be used to assess quality in health sciences education.

An important competence in medical and health sciences education is professional problem solving or clinical reasoning. Experts use varying approaches in clinical reasoning, either analytical or non-analytical, or both [12]. Script concordance testing has been used to assess clinical reasoning [13–15] and to trace students’ progression in the development of clinical reasoning [16]. Students need training in clinical reasoning [12], and verbalisation of reasoning processes forms an important part of such training [17]. Authentic clinical cases are often used for learning clinical reasoning and methods exists for structuring such discussions, for example problem-based learning [18, 19]. The case method, originally developed at Harvard Business School [20] has been adapted to medical and health sciences education and used for student active learning [21–24]. The students are involved in discussing a case using a structure that closely resembles the clinical reasoning process [17].

The aim of this study was to monitor and describe students’ progression in professional clinical reasoning skills during health sciences education using observations of group discussions following the case method.

## Methods

### Study design

This study had an observational design using a qualitative method of analysis. A modified Harvard case method [23] was used as a tool for teaching and learning and rubrics [25] as a tool for identifying clinical reasoning skills.

### Context and setting of the study

In Sweden, outcomes for higher education are specified in the Higher Education Ordinance [26]. Swedish higher education is modular; the students pass through a series of courses and each course requires separate assessment, with no final mandatory graduation examination. The faculty of medicine at Lund University has several health education programmes including occupational therapy (OT), speech-language therapy (SLT) and midwifery (MW). These programmes took part in this study after an invitation to teachers who applied the case method in their teaching. The OT programme is a three-year undergraduate programme, while the SLT programme has undergraduate intake leading to an exam at advanced level after 4 years. The MW programme is 1.5 years at advanced level with nurse qualification (3 years) as entry requirement. All three programmes include clinical placements.

### Procedure

The study was carried out in year 2014-2015. A modified Harvard case method [23] was used to monitor progression across years through the professional programmes that took part in the study. The students were required to discuss and proceed through six steps, following this structure, c.f. Levett-Jones [17].

1. Identification of the problem that a professional faces
2. Identification of relevant facts
3. Discussion of what could happen if left unattended
4. Suggestion of actions by professional to solve the problem
5. Analysis of potential results of suggested actions
6. Evaluation of actions

If relevant, students should apply an holistic perspective including perspectives from other professions as well as ethical, legislative and financial aspects when discussing the case. This process resembles the strategies suggested by Klein as important aspects of expertise for decision-making skills [27].

The discussion started when the students were presented with a case written in a narrative style in the perspective of a professional from the future profession of the students. This professional faces a challenge, concerning a patient or a client. The cases were authentic, taken from real-life experience, and open-ended in the sense that differing potential solutions should be possible [24]. The cases differed in detail and length depending on the clinical situation. For example, the case used by the midwifery students described a scenario during the course of a day, but the case in occupational therapy covered a situation developed during a year or longer. The teacher in the case method had the role of a facilitator who guides the students through the structure (see above). Ten to–24 students took part in each case method session (Table 1). They worked in small groups (4–5) in the same room with intermittent discussions in the large group. Facilitation by the teacher took place only in the large group. A whiteboard was used to document work in the large group, whereas the small groups documented their work in notes. The whiteboard was sectioned and headlines (the six step presented above) were used to guide the students’ discussion [22]. The whole session took 1-2 h depending on the complexity of the case.

**Table 1:** Numbers of participating students in cohorts, number and qualifications of observers and duration of observations in this study

Programme	Students	Observers	Duration of observation
OT 3 years undergraduate entry	Year 1: 10 Year 2: 10 Year 3: 10	3 teachers (reg. Occupational therapists, PhDs)	Two hours per group
SLT 4 years undergraduate entry	Year 1: 24 Year 2: 24 Year 3: 24 Year 4: 10	2 teachers (reg. Speech-language therapists, PhDs)	One hour per group
MW 1.5 years graduate entry	Year 1: 20 Year 2: 16	3 teachers (reg. Midwives / PhDs; 1 educational developer, professor)	One and a half hours per group

OT = occupational therapy; SLT = speech-language therapy; MW = midwifery

In this study, it was a prerequisite that the same case was used in all student cohorts within each professional programme. Special comprehensive cases were developed for the study. Since the three professions differ in area of responsibility, all three cases were prepared in order to capture profession-specific clinical reasoning skills. The OT case concerned a client-centred, occupation-based intervention, including the individual, family, community and organizations. The case had a high complexity in order to challenge students throughout the programme. The SLT case addressed speech and language impairment in the school years, requiring knowledge of aetiology and diagnostics, as well as long-term educational consequences, professional delineations, and inter-professional collaboration. The MW case concerned the complexity of progress during normal labour, which is a very central problem for the midwifery profession and might be met by any student or recently graduated midwife.

### Participants

Students in all years of the three programmes were invited to take part in the study and informed that the observations were concerned with the groups, not with individuals. In the OT programme students from all 3 years were invited to participate voluntarily in the case discussions after a compulsory seminar. In the SLT-programme four cohort of students were invited to participate and in the MW two cohorts of students representing first and third semester of the programme. In the SLT year 1-3 and MW programmes the case method sessions were scheduled in the regular courses since they were considered to also be learning opportunities for the students. Students who did not wish to participate could be given the case for self-study, but all invited students accepted to participate. In year four of the SLT programme where the students were performing their degree projects, case sessions could not be scheduled. Students were invited to voluntarily take part in a case discussion. Table 1 includes details of the number of students and cohorts in the study.

### Observation

Two or three observers observed the students (Table 1), while taking part in a discussion of a case during a case method session as described above. In addition, a teacher, well acquainted with the case method, was present as a facilitator who guided the process but intervened as little as possible. Since observations of students of all cohorts in a single programme were carried out during the same period, the students belonged to different cohorts.

A rubric to be used as a tool for observations was developed within a larger project including the faculties of engineering and economics [25]. In Sweden there are generic learning outcomes for all Higher Education irrespective of discipline [26] and the rubric was developed in accordance with these to be used to when the case method was used for teaching and learning. The rubric was tested with students from engineering; this study will be reported elsewhere*.*

The final version of the rubric had four dimensions:

1. Problem-solving process (*Identification of the problem; Use of data in the case; Analysis; Synthesis and decision*)
2. Disciplinary knowledge (*Professional language; Prior knowledge*)
3. Character of the discussion (*Theory-based; Polemical; Supportive; Perspective-shifting/metaphoric*)
4. Communication (*Communication within the group; Trust within the group; Interaction with the teacher*)

Point for observation were described and outlined in the rubric. All observers were instructed, but not formally trained, in using the rubric by observing the four dimensions. The rubric was used as a tool for observation of group discussions and observers added written notes in a column under each dimension. Details about observers are included in Table 1. The observers were all teachers at the medical faculty, well trained in observing group discussions and well familiar with the case method. All students were familiar with case-discussions and were informed about the study aim and that their discussions would be observed but not informed about the rubric’s content. It was clarified that it was not an individual summative assessment. After the observation, the students were informed about the rubric and they had opportunity to ask questions.

### Data analysis

The observers used the rubric in order to employ the same standardized procedure when observing the different cohorts and programmes. Results were compiled for each cohort and programme. The analysis started with comparison of the written notes from each observer collected during the case method sessions. All data were reviewed for content and coded for correspondence to the four dimensions in the rubric using a deductive content analysis [28]. Consensus on description in each rubric for all sessions in the respective programme was achieved through mutual discussions between the observers until agreement was achieved. Trustworthiness [29] was supported by the observers’ active participation in every phase of the analysis process, including the preparation, organization and interpretation of data.

## Results

The results from this qualitative observational study describe students’ progression in problem solving process, disciplinary knowledge, character of discussion and communication, observed during the case method sessions. They are summarized for each participating programme separately both in the text below and in Table 2.

**Table 2:** Summary of findings presented for each programme and cohort

Dimensions	Occupational Therapy (OT)	Speech-Language Therapy (SLT)	Midwifery (MW)
Problem-solving process			
Identification of the problem	Y1 students identified the main problem but focused on details related to medical issues and motor function. Y2 students identified a range of problems related to the individual and the main carer. Y3 students were able to recognize the added importance of contextual factors.	Y1 students identify the problem quickly and informally, relating the information to personal experience. Y2 use a problem-based approach, trying out hypotheses on each other. Y3 and Y4 students evaluate explanations by alternating between roles (SLT, caregiver, school personnel).	Y1 students had difficulties in identifying the main problem whereas Y2 students identified relevant problems in a structured way
Use of data in the case	Y1 students needed support from the facilitator to identify the relevance of some key information. Y2 students used profession-specific models to identify and discuss relevant information. Y3 students identified relevant information in the case without the need for structured tools.	All information is considered in the discussions by Y1 to Y4.	Some Y1 students missed some information given and wanted more. Y2 students identified and used relevant information in the case.
Analysis	Y1 students described and analysed primary motor and psychological factors that influenced performance. Y2 students demonstrated a deeper understanding by including basic environmental factors. Y3 students showed understanding of key components of occupational performance and participation in the community. They moved beyond home environment and began to consider participation, identity and societal pressures.	Y1 to Y4 students use course content and experiences from clinical training to guide their analyses. Y1 also include personal experiences in the analysis, relating to friends and family members in a situation similar to the one described in the case. Y3 and Y4 make multifaceted analyses, relating to intrapersonal and organizational domains.	The analysis made by Y1 students was superficial and some groups identified lack of knowledge. Y2 students systematically made a deep analysis.
Synthesis and decision	Y1 students used occupational therapy theories but a lack of knowledge to support intervention. Y2 students identified a need for additional information and teamwork. Y3 students based their decisions on relevant theories and frames of references to support evidence-based intervention.	Y1 suggest many approaches to address the issue, based on interviews with clinical SLTs and common knowledge of the SLT role. Y2 show clear signs of their future role, with descriptions of suggested assessments and intervention. Y3 and Y4 present individualized interventions for parents and school personnel.	Y1 students had difficulties; they needed more knowledge and advice from a facilitator and focused more on caring than midwifery. Y2 students arrived at decisions based on professional competence.
Disciplinary knowledge			
Professional language	Y1 students used informal language but made an effort to keep an occupation-focused approach. Y2 students used correct concepts connected to theories, whereas Y3 students used various frames of references and ethical dilemmas in their discussions	Y1 students mainly use colloquial language, but show basic understanding of advanced scientific and clinical concepts. Y2 students replace lay definitions with scientific equivalents to guarantee mutual understanding. Y3 and Y4 students have acquired professional concepts and vocabulary.	Students in both cohorts used correct terms and concepts; Y1 students sometimes used informal language whereas Y2 students used the language of the maternity ward.
Prior knowledge	Y1 students used their knowledge in how constraints in motor skills may affect occupational performance. Y2 and Y3 expanded this and also used their understanding of the Person-Environment-Occupational Performance model in their clinical reasoning. Y3 students used ethical principles, social justice and laws related to OT.	Y1 is baseline, with previous course content in related subjects only and no SLT courses. Y2 and Y3 have completed courses in atypical child language development and clinical training, relevant to the case, with Y3 also having additional research methodology. Y4 also had completed a course in reading disability, and clinical training, relevant to the case.	Y1 students had deep knowledge in caring but superficial in midwifery. Y2 students had relevant experience-based professional knowledge.
Character of the discussion			
Theory based	Y1 students shared/compared their theoretical knowledge. Y2 students showed adequate knowledge and discussed consequences whereas Y3 students showed theoretical understanding and supported evidence-based practice.	Y1 mainly retold the case, explaining with basic theoretical constructs. Y2 and Y3 showed evolved theoretical understanding and ability to interpret and explain. Y4 could identify gaps in their knowledge, and demarcations to related professions.	Y1 students had theoretical knowledge, but insufficient. Y2 students had adequate knowledge and referred to experience, not to literature
Polemical	Y1 students discussed, came to consensus and agreed, Y2 students discussed and challenged one another. Y3 students were able to deal with challenges, resolve them and reached agreement on resources needed to solve the case.	Y1 showed great consensus, with students filling in the comments of others. Y2 corrected each other, and asked for additional information and references to arguments and standpoints. An argumentative tone in Y4, with provocative questions, stimulated further discussions.	Y1 students largely agreed. Y2 students sometimes disagreed, in a kind manner.
Supportive	Y1 students supported each other well. Y2 and Y3 students both supported and challenged learning in the group.	All students, Y1 to Y4, had a supporting discussion climate, with questions aimed to reach a common goal rather than exposing gaps in the knowledge of others.	Students were supportive in both groups an in Y2 even reinforcing.
Perspective-shifting/metaphoric	Y1 students went back to the case and found important dilemmas they first missed. Y2 and Y3 students added perspectives and discussed “outside the box”. Including an interprofessional teamwork focus.	Y1 advanced the discussion by taking the perspective of the patient, parents and school personnel, but not the SLT. Y2, in contrast, showed few examples of looking beyond the SLT role. Y3 and Y4 included all perspectives in their discussions, and those of other professionals, with a focus on the SLT role.	In both cohorts by returning to the case and finding new perspectives.
Communication			
Communication within the group	Y1 students discussed and listened well, Y2 students talked and questioned each other and Y3 students communicated in a more creative manner, generating new ideas and professional thinking.	The discussions were conducted increasingly independent of the facilitator from Y1 to Y4.	In both cohorts students communicated well: talked, listened and asked questions. Y2 students exchanged experiences and could teach each other in a kind manner.
Trust within the group	Friendly, trustfully and open in Y 1 and 2. Some Y3 students were first eager to use and show their knowledge but after a while all students had the opportunity to raise their ideas and comments.	In all groups, Y1 to Y4, students appear comfortable with each other, although a few students remain quiet in the full-group discussions. A relaxed atmosphere in Y4 allowed playful comments without losing a professional tone.	There was trust and openness in both cohorts. The attitude was professional but more friendly and careful in the Y1 group.
Interaction with the teacher	Y1 students needed the facilitator more than Y2 students who asked some questions. Y3 students discussed with each other and were in charge of the process and decisions taken.	Y1 and Y3 had much to say but needed questions from the facilitator to start the discussions while Y2 and, in particular, Y4 worked more independently.	The small groups worked independently. Y1 students needed a lot of help from the teacher when all groups gathered to exchange experiences, whereas the Y2 students took care of most of the discussion themselves also during the gathering.

Y1 = year 1; Y2 = year 2; Y3 = year 3; Y4 = year4

### Occupational therapy programme

In the OT programme students were recruited from three cohorts; first, second and third year. We found clear differences in the problem-solving process and how the students discussed the case depending on study year. Disciplinary knowledge was observed and students in the first year did not use a professional language or concepts specific for a family-centred setting. However, they used an occupation-focused approach in their discussions. It was also evident that students in the first year made an effort to understand the impairments on body function and body structure level before they started to discuss occupational performance skills. They identified the problem but needed cues from the facilitator to grasp the complexity of the case. Some obvious connections were made, but to some degree, there was a lack of important understanding of how individual, family, community and environmental factors impact on occupational performance possibilities. The students in the second year used a mature professional language and tried to use correct terminology. They identified key roles of families, peers and communities as factors influencing on occupational performance. The case was thoroughly analysed and relevant assessment tools discussed. Some appropriate interventions were mentioned, although a lack of collaboration with other professionals was evident. The character of the discussions and communication was identified; students worked in a constructive way and gave comments and feedback to each other in the groups. Students in the third year identified the key problem by using mature and flexible teamwork. They discussed in a broader context such as autonomy, ethics and human rights. The complexity in the case was identified using competencies within the group. Primarily they addressed occupational performance and discussed challenges relevant to the problem. Key policies were considered, such as social security systems, health policies, social justice and human rights. Students in this group used a professional language and prior knowledge connected to theory. The students made reflections beyond the case presented and showed an understanding of a relevant approach to interventions.

### Speech-language therapy programme

Four groups of students were recruited from the SLT programme, representing all 4 years of the programme. The groups of students differed regarding depth of knowledge and level of reasoning. First-year students showed limited disciplinary knowledge as evidenced by a high degree of colloquial language, resulting in less clearly defined concepts and requests for clarification from other students. They showed basic knowledge of scientific and clinical concepts but these were more fully mastered by second-year students, who showed a more developed problem-solving process, arriving more quickly at a common identification of the problem through hypothesis testing. The character of the discussion of the students in their third and fourth years of studies showed evidence of a deeper and more advanced level of reasoning, with students alternating between the perspectives of the SLT, patient, caregiver and school personnel. While students from all years relied heavily on previous course content to guide their analyses, first-year students also let personal experiences and anecdotal evidence influence their interpretation. In contrast, in later years, statements without clear references were questioned by fellow students. Year-four students asked provocative questions to promote the discussion, without losing a professional conversational tone. The communication of all groups showed high levels of independence from the facilitator, who only occasionally, primarily in the first year, was required to guide the discussion with questions and comments. A noteworthy transition from a more specialized competence to a team-based perspective was observed. Students in the third year quickly identified problems and the SLT role in resolving the issue. Students in the fourth year were reluctant to make similar interpretations, and were more prone to a team-based solution, acknowledging the competence of other professionals, in particular school personnel.

### Midwifery programme

Two groups of students were recruited in this programme, representing the first and second years of the three-semester programme. We found progression in the problem-solving process in the way the students discussed and analysed the case, identifying the problem, using information and decision-making in the group. First-year students had difficulties in identifying the main problem and instead discussed several problems as though equally important. When using information in the case, students from first year read all the facts before discussing, whilst second-year students started their discussion quickly, without reading all the facts. However, they returned to the case during the discussion to gather more facts. First-year students’ decision-making varied but was mainly tentative based on theoretical knowledge. Students in the second year made and integrated decisions based on professional knowledge and experience and evidence was not specifically alluded to. The disciplinary knowledge also clearly progressed in the use of professional language, concepts and terms and the use of prior practical experience. A striking difference was that first-year students used theory-based knowledge from theoretical courses and their experience as nurses, whereas second-year students used experience-based knowledge from midwifery practice. First-year students also exhibited some difficulties in using the problem-solving model whereas second-year students dealt with central problems using the problem-solving model spontaneously. All students used professional concepts and terms according to course literature, and second-year students in addition communicated in the same effective and relaxed way as professional midwives. The main difference between the two groups, when they discussed and solved the problem, concerned practical experience. When first-year students discovered a lack of knowledge hampered progress in the discussion, they turned to the course literature for help. They used theoretical knowledge that at times was insufficient. Second-year students seemed to have a clear theoretical grounding, even though they did not refer to the literature but rather to practical experiences. All groups discussed in a supportive trusting way and also listened to and considered each other’s experiences. However, second-year students were shifted perspective more in the discussions. First-year students interacted more with the teacher whereas second-year students did not seem to need the teacher in their discussion.

## Discussion

We have found it is possible to monitor progression by using the case method and rubrics as tools for identifying clinical reasoning. The results reveal the students’ transition over time from strictly theory-based knowledge to a reasoning ability characterized by clinical considerations and experiences when trying to solve the clinical problem. This is also in line with previous findings by Wahlgren and Ahlberg [11].

In the problem-solving process, students in the early stages of the programme had more focus on and questions to the facilitator than students had in later stages of the programmes, who were more secure and confident. When identifying the relevant problem, first-year students had a somewhat fragmented approach to the case, reflected in the identification of several problems and an inability to identify the most relevant problem. For example, students early in the OT programme relied more on learning from the anatomy and neurological courses in an atomistic way and had difficulties integrating this in a more holistic way. Marton et al. [30] describe atomistic learning as fragmented and deep learning as holistic, where the student strives to understand meaning, connection, context and implication. Later in their programmes, students were more confident in their application of knowledge and had clinical experience to identify the most relevant problem(s) in the case efficiently. The transition from a fragmented approach to a holistic approach could be observed in all programmes. It has previously been shown in a study about midwifery students’ written reflections that their knowledge moves from a fragmented to a holistic approach during their education [31]. Perhaps the case method can inspire students and support deep learning early in the education.

A linear relationship between nursing student’s scores on a script concordance test and their experience of clinical practice was shown by Dawson et al. [13]. Using a progress test Williams et al. [16] also found a steady increase over the study years in clinical reasoning skills among medical students. A test with many questions may have a better reliability for an individual student, but observations like the ones we have used provide opportunities to study students’ development on group level.

We observed an insecurity amongst first-year students when they discovered that their knowledge was not sufficient, made evident by the use of textbooks, relying on personal and anecdotal evidence or looking for interaction from the teacher/facilitator. Vocabulary and use of professional concepts and terms developed over the cohorts. First-year students used layperson language influenced by textbook knowledge. Students who had clinical experience used a professional language, similar to the language used when qualified professionals communicate (as testified by the observers). Jones et al. [32] point out that standardized language is very important as an effective strategy to clarify professional nursing practice, which is equally important for all professionals. A precise vocabulary enables the formulation of precise questions, which receive focused answers, as shown in the present study by the prompt arrival at a clear identification of the relevant problems by 3rd and 4th year students. In addition, fourth-year students appeared more familiar with the discussion format, and asked provocative and challenging questions to fellow students, in order to advance the discussion.

Multiple-choice tests to measure progress by quantitative means have shown a steady increase in the knowledge of medical students [33]. Such tests are more reliable than case-based tests mainly due to better sampling [8]. This study was performed using one single case per programme, which may have compromised reliability, due to case specificity. However, it has been shown that generic skills contribute to clinical performance [34] and the case specificity has been questioned [35]. A combination of methods is probably preferable to obtain both reliable results concerning students’ knowledge as well as assessing generic skills. To increase the reliability of observations a rubric can be used, preferably complemented with examples [36] as was done in this study. Assessor training could further have increased the reliability of marking [37]. However, experts have been shown to have a high degree of agreement on the key elements of the clinical reasoning process [38]. The characteristics of communication in all programmes were distinguished by trust, most obvious in the later years. Students in the two undergraduate programmes (OT, SLT) used a team-based approach to problem solving in their final years. SLT students in their third year of studies quickly and accurately identified the necessary contributions of the SLT in addressing the issue described in the case. Fourth-year students, in contrast, acknowledged the necessity of a team-based approach, in which the plan of action was determined in close collaboration with other health and education professionals. Second-year students in the OT programme were observed as having the personal, professional and interprofessional skills that represent an isolated specialist. In their final year, this had changed to the competence of a team member who requires the expertise of other professionals for a broader understanding of challenges described in the case. This was not observed for MW students, most likely reflecting the midwife’s independent professional role with only an auxiliary nurse in a small team in the given case. Communication skills are important for teamwork in the future profession of the students [39]. Developing competence in communication during education of health professionals has been identified as being of major importance for healthcare by the WHO [40] and by a recent Lancet commission [41].

The use of only theoretical knowledge in the first year students could be compared to the reliance on rules in the novice stage in the Dreyfus model [42]. Novices follow rules whereas experts through experience have developed more intuitive and holistic ways of solving problems [42, 43]. The more fluent and precise identification of the relevant problem in later years could perhaps be interpreted as the development of more intuitive thinking.

In all programmes students’ development in problem identification and clinical reasoning were influenced by clinical training and professional reasoning. Professional reasoning includes important learning of values, attitudes and beliefs of the profession [44]. Despite the positive effects, there also can be a potential risk with professional socialisation [45] and it has been argued that there is a risk that professional socialisation may have negative consequences. The new professionals may prefer to do things in the way they always have been done, rather than practise the latest evidence. It is important for both students and teachers to be aware of this challenge.

We observed a developing reliance on clinical experience in the students’ discussions and at the same time an integration of theoretical knowledge and a development of an holistic perspective. This is in line with the two-dimensional model of professional development suggested by Dall’Alba and Sandberg [46]. They add a second dimension to skill progression, namely embodied understanding of practice, allowing for differences between individuals’ development trajectories. The students showed a progression of problem-solving skills, but also, and perhaps more prominently, a progression in embodied understanding of practice.

The case method used in the present study shows potential to track the progression of students learning during education. Without the need for formal assessment, it can provide the teacher with continuous information on students’ level of knowledge and reasoning, necessary to adjust instructions and the amount of support. The importance of accord between course learning outcomes, assessments and integration of different parts of the course content for student learning, has been proposed by Biggs [47] and termed constructive alignment.

### Strengths of the study

Studies of this kind might be easily integrated into regular teaching and students could learn from the observed sessions making participation of value to the students. Using the case method enabled the application of and to describe students clinical reasoning skills. Another strength is that the same rubric was used for all observations. This means that the results could easily be compared across programmes. Further, all observers except one were qualified professionals with clinical experience. Small groups of students discussing the cases were observed as recommended by Benson et al. [48] since it allows for closer evaluation of the group process.

The strategy to enhance trustworthiness of the content analysis was to reach high intercoder reliability [28] between the observers. The rubric was developed prior to the study and all observers were familiar with the domains observed. Throughout the data collection and during the deductive content analysis observers (researchers) discussed the coding scheme used in the analysis process.

### Weaknesses of the study

A weakness of the study is that the case sessions were scheduled as part of regular teaching for some cohorts, and as voluntary extracurricular activity for some. The students who volunteered may not be representative of the whole cohort. The groups of students observed were small and we cannot exclude a possibility that there can be variations between different cohorts. The influence of individual students could be strong in such small groups, and we cannot exclude large variations between individual students’ progression. Students worked in teams and the competence in the team might be higher than the competence of the individual student. We have observed structured discussions of a single case for each profession and the structure and the context of the case could have influenced the result. All students had experience of the case method. However, students in later years were more experienced in using the case method and this could also have influenced the results. Another weakness is that the observers were not formally trained in how to observe. They were all teachers who had used the case method in their teaching but they were new to this rubric. Using a rubric may also result in other important information being lost. The use of a generic rubric (a consequence of taking part in a university-wide project) could be seen as a weakness. However, we believe that the rubric, though generic, covers relevant aspects of health sciences clinical reasoning well). Further, since the observers were teachers in the programmes they knew which cohort they observed and this can have caused a bias in the observation.

This study relies exclusively on verbal expression of professional skills. These are important as such, but professional competence also involves action in practice. Students’ prior knowledge, norms and values may be both a strength and weakness.

## Conclusions

Our observations revealed progression in several aspects of students’ clinical reasoning skills on a group level in their discussions of clinical cases. Observing students’ discussions of professional cases could be used to evaluate progression and quality in health sciences education. We have found it is possible to monitor progression by using the case method and rubrics as tools for identifying clinical reasoning. This can be considered as an evaluation of quality of curriculum, which is important in higher education. In addition, it can be considered resource saving to use an already existing learning tool, the Case Method, for the purpose of evaluation.

## Abbreviations

MW: Midwifery
OT: Occupational therapy
SLT: Speech-language therapy
WHO: World Health Organisation
